# Application of hepatic cytochrome *b*_*5*_/P450 reductase null (HBRN) mice to study the role of cytochrome *b*_*5*_ in the cytochrome P450-mediated bioactivation of the anticancer drug ellipticine

**DOI:** 10.1016/j.taap.2019.01.020

**Published:** 2019-03-01

**Authors:** Lindsay Reed, Radek Indra, Iveta Mrizova, Michaela Moserova, Heinz H. Schmeiser, C. Roland Wolf, Colin J. Henderson, Marie Stiborova, David H. Phillips, Volker M. Arlt

**Affiliations:** a Department of Analytical, Environmental and Forensic Sciences, MRC-PHE Centre for Environment and Health, King's College London, London, United Kingdom; b Department of Biochemistry, Faculty of Science, Charles University, Prague, Czech Republic; c Division of Radiopharmaceutical Chemistry, German Cancer Research Center (DKFZ), Heidelberg, Germany; d Division of Cancer Research, Jacqui Wood Cancer Centre, School of Medicine, University of Dundee, Ninewells Hospital, Dundee, United Kingdom

**Keywords:** Cytochrome P450, Cytochrome *b*_*5*_, Mouse models, Metabolism, DNA Adducts, CYP, cytochrome P450, Cyb5, cytochrome *b*_*5*_, Cyb5R, cytochrome *b*_*5*_ reductase, EROD, 7-ethoxyresorufin *O*-deethylation, HBRN, Hepatic Cytochrome *b*_*5*_/P450 Reductase Null, HRN, Hepatic P450 Reductase Null, HPLC, high performance liquid chromatography, MROD, 7-methoxyresorufin *O*-demethylation, NADH, nicotinamide adenine dinucleotide, NADPH, reduced nicotinamide adenine dinucleotide, PA, phenacetin, POR, NADPH:cytochrome P450 oxidoreductase, TLC, thin-layer chromatography, WT, wild-type

## Abstract

The anticancer drug ellipticine exerts its genotoxic effects after metabolic activation by cytochrome P450 (CYP) enzymes. The present study has examined the role of cytochrome P450 oxidoreductase (POR) and cytochrome *b*_*5*_ (Cyb5), electron donors to P450 enzymes, in the CYP-mediated metabolism and disposition of ellipticine *in vivo*. We used Hepatic Reductase Null (HRN) and Hepatic Cytochrome *b*_*5*_/P450 Reductase Null (HBRN) mice. HRN mice have POR deleted specifically in hepatocytes; HBRN mice also have Cyb5 deleted in the liver. Mice were treated once with 10 mg/kg body weight ellipticine (*n* = 4/group) for 24 h. Ellipticine-DNA adduct levels measured by ^32^P-postlabelling were significantly lower in HRN and HBRN livers than in wild-type (WT) livers; however no significant difference was observed between HRN and HBRN livers. Ellipticine-DNA adduct formation in WT, HRN and HBRN livers correlated with Cyp1a and Cyp3a enzyme activities measured in hepatic microsomes in the presence of NADPH confirming the importance of P450 enzymes in the bioactivation of ellipticine *in vivo*. Hepatic microsomal fractions were also utilised in incubations with ellipticine and DNA in the presence of NADPH, cofactor for POR, and NADH, cofactor for Cyb5 reductase (Cyb5R), to examine ellipticine-DNA adduct formation. With NADPH adduct formation decreased as electron donors were lost which correlated with the formation of the reactive metabolites 12- and 13-hydroxy-ellipticine in hepatic microsomes. No difference in adduct formation was observed in the presence of NADH. Our study demonstrates that Cyb5 contributes to the P450-mediated bioactivation of ellipticine *in vitro*, but not *in vivo*.

## Highlights

• The anticancer drug ellipticine is activated by cytochrome P450 (CYP) enzymes.
• Cytochrome *b*_*5*_ (Cyb5) and NADPH:CYP oxidoreductase (POR) modulate CYP enzyme activity.
• Ellipticine bioactivation was investigated in mouse lines with hepatic deletions of Cyb5 and/or POR.
• POR strongly contributed to ellipticine bioactivation *in vivo*.
• Cyb5 contributed little to ellipticine bioactivation *in vivo* contrasting previous *in vitro* studies.

## 1 Introduction

Ellipticine (5,11-dimethyl-6*H*-pyrido[4,5-*b*]carbazole) is a cytotoxic alkaloid isolated from the *Apocynaceae* family of plants. Both ellipticine and its derivatives possess anti-HIV and anti-tumour properties allowing it to be used against several cancers with limited toxic side effects and no haematological toxicity by functioning through multiple mechanisms that result in cell cycle arrest and initiation of apoptosis (Martinkova et al., 2010; Stiborova et al., 2011; Miller and McCarthy, 2012; Stiborova and Frei, 2014). The main mechanisms by which ellipticine exerts its anti-tumour, cytotoxic and mutagenic effects are inhibition of topoisomerase II, intercalation into DNA and enzyme-mediated formation of covalent ellipticine-derived DNA adducts (Garbett and Graves, 2004; Stiborova and Frei, 2014; Banerjee et al., 2015; Vann et al., 2016).

**Fig. 1:**
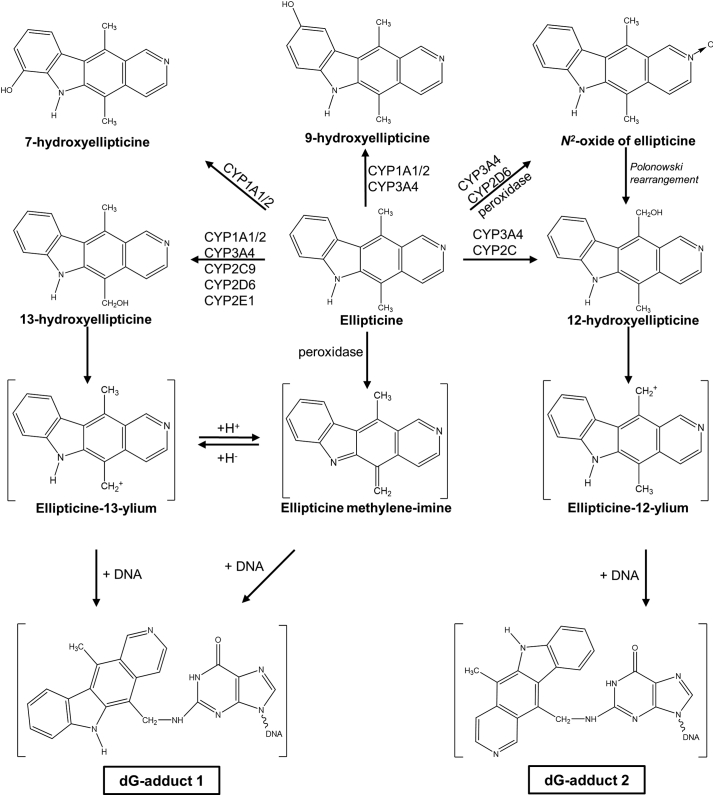
Pathways of biotransformation and DNA adduct formation of ellipticine catalysed by P450 and peroxidase enzymes showing the identified metabolites and those proposed to form DNA adducts. The compounds shown in brackets have not been previously detected under experimental conditions and/or not structurally characterised.

Ellipticine needs to be metabolised to exert its pharmacological effects. These enzyme-catalysed reactions also generate detoxication products leading to the excretion of the drug. Activation of ellipticine (*i.e.* generation of pharmacologically active metabolites) is catalysed by cytochrome P450 (CYP) enzymes and peroxidases, which generate reactive intermediates capable of damaging DNA by forming covalent adducts (Stiborova et al., 2001; Stiborova et al., 2003; Stiborova et al., 2003; Stiborova et al., 2007; Stiborova et al., 2007; Stiborova et al., 2008; Stiborova et al., 2012; Stiborova et al., 2012). As shown in Fig. 1, ellipticine is oxidised by P450 enzymes to form five metabolites, including the reactive metabolites 12-hydroxy- and 13-hydroxy-ellipticine which dissociate to ellipticine-12-ylium and ellipticine-13-ylium and bind to DNA (Stiborova et al., 2004; Aimova et al., 2007; Stiborova et al., 2014; Stiborova and Frei, 2014; Stiborova et al., 2014). The *N*^2^-oxide is also considered an active ellipticine metabolite as it converts to 12-hydroxy-ellipticine by the Polonowski rearrangement (Stiborova et al., 2004; Kotrbova et al., 2006). 7-Hydroxy-ellipticine and 9-hydroxy-ellipticine are considered detoxication metabolites due to their efficient excretion by experimental animals (Stiborova et al., 2012; Stiborova et al., 2012). Therefore, understanding the role of P450 enzymes in ellipticine metabolism is important both pharmacologically and toxicologically.

A number of transgenic mouse lines (*e.g. CYP*-knockout or *CYP*-humanised) have been applied to study the contribution of individual P450 enzymes to chemical-induced genotoxicity and carcinogenesis (Stiborova et al., 2012; Nebert et al., 2013; Reed et al., 2018). However, due to the large number of CYP enzymes it has been difficult to determine the *in vivo* role of P450 enzymes as a whole as there are overlapping substrate specificities. We have used the Hepatic P450 Reductase Null (HRN) mouse model in order to overcome this limitation (Arlt et al., 2015). HRN mice have a deletion of NADPH:cytochrome P450 oxidoreductase (POR), the predominant electron donor to P450s, specifically in their hepatocytes (Henderson et al., 2003). This deletion results in the loss of essentially all hepatic P450 activity and the mice have been used to investigate hepatic *versus* extra-hepatic P450 mediated metabolism of several carcinogens including ellipticine (Arlt et al., 2005; Stiborova et al., 2008; Levova et al., 2011; Arlt et al., 2012). HRN mice formed 65% lower levels of ellipticine-DNA adducts in their livers than wild-type (WT) mice, demonstrating the importance of P450 activity in the hepatic bioactivation of ellipticine (Stiborova et al., 2008).

Although POR is viewed as the predominant electron donor to P450 enzymes, cytochrome *b*_*5*_ (Cyb5) can also act as the electron donor (Yamazaki et al., 2002; Finn et al., 2008). Cyb5 can modulate P450 activity in three ways: (*i*) by direct transfer of both electrons *via* cytochrome *b*_*5*_ reductase (Cyb5R) in a pathway independent of POR (Yamazaki et al., 1996; Yamazaki et al., 1996); (*ii*) by transfer of the second electron from either POR or Cyb5R (Zhang et al., 2007); or (*iii*) by acting as an allosteric modifier of the enzyme in a non-catalytic role that can enhance reactions for many, but not all, P450 enzymes (Yamazaki et al., 2002). Cyb5 is both substrate and enzyme specific, and has been shown to both stimulate and inhibit P450 reactions, making it difficult to predict the contribution of Cyb5 to xenobiotic metabolism.

Previous studies using reconstituted systems investigated the role of Cyb5 in the metabolic activation of ellipticine *in vitro* showed that the presence of Cyb5 resulted in a considerable increase in the activation metabolites 12-hydroxy- and 13-hydroxyellipticine (Kotrbova et al., 2011; Stiborova et al., 2012; Stiborova et al., 2012; Stiborova et al., 2017). The formation of ellipticine-DNA adducts was also shown to increase ~6-fold in the case of CYP1A1, ~4-fold for CYP1A2 and ~3-fold for CYP3A4 (Kotrbova et al., 2011; Stiborova et al., 2012). These findings were supported by studies using human recombinant P450s in Supersomes™ with CYP3A4 and 1A1 being the most efficient at forming ellipticine-DNA adduct 1 and with adduct 2 being formed by CYP2C19, 2C9 and 2D6 in the presence of Cyb5 (Stiborova et al., 2012). Rats exposed to ellipticine have also shown a significant increase in the expression of both Cyb5 mRNA and protein, and hepatic microsomes isolated from these rats catalysed ellipticine oxidation more efficiently (Stiborova et al., 2016). Together these studies provide evidence for the role of Cyb5 in the bioactivation of ellipticine both *in vitro* and *in vivo*.

Hepatic Cytochrome *b*_*5*_/P450 Reductase Null (HBRN) mice (Henderson et al., 2014) lack both POR and Cyb5 in their livers and have reduced P450 activity relative to HRN mice (Henderson et al., 2013). In the present study we have used both the HRN and HBRN mouse lines to investigate the contribution of Cyb5 to the metabolic activation of ellipticine to form DNA adducts *in vivo* alongside microsomal incubations to investigate metabolite and DNA adduct formation *in vitro.* Hepatic microsomal P450 enzyme activity and protein expression have also been assessed.

## 2 Materials and methods

### 2.1 Chemicals

Ellipticine, NADH (as disodium salt; purity ~95%), NADPH (as tetrasodium salt; ~98% purity), Sudan I and 7-methoxyresorufin were obtained from Sigma Chemical Co (St Louis, MO, USA). Testosterone and 6β-hydroxytestosterone were purchased from Merck (Darmstadt, Germany).

### 2.2 Animal treatment

All animal experiments were carried out at the University of Dundee under licence in accordance with the Animal (Scientific Procedures) Act (1986), as amended by EU Directive 2010/63/EU, and with local ethical approval. HRN (*Por*^*lox/lox*^/*Cre*^*CYP1A1*^*)* mice and HBRN (*Cytb*_*5*_^*lox/lox*^/*Por*^*lox/lox*^ ± *Cre*^*ALB*^) mice on a C57BL/6 background were derived as described previously (Henderson et al., 2003; Henderson et al., 2013). Animals were maintained in open-top cages, with free access to food (RM1 diet, Special Diet Services, Essex, UK) and water, and a 12-h light/dark cycle. Mice homozygous for the floxed *Por* locus (*Por*^*lox/lox*^) were used as wild-type (WT). Groups of female HRN, HBRN and WT mice (3 months old, 25–30 g) were treated intraperitoneally (i.p.) with 10 mg/kg body wt ellipticine (*n* = 4/group) based on a treatment regimen used previously in HRN mice (Stiborova et al., 2008). Ellipticine was administered dissolved in 1% acetic acid at a concentration of 2.5 mg/ml. Control mice (*n* = 3/group) received the solvent only. Animals were killed 24 h after the single dose and their tissues (liver, lung, small intestine, kidney, spleen, bladder and colon) were collected, snap-frozen and stored at −80 °C until analysis.

### 2.3 Ellipticine-DNA adduct detection by ^32^P-postlabelling analysis

Genomic DNA from whole tissue was isolated by a standard phenol-chloroform extraction method and DNA adducts were measured for each DNA sample using the nuclease P_1_ enrichment version of the thin-layer chromatography (TLC)-^32^P-postlabelling method as described previously (Stiborova et al., 2008; Willis et al., 2018). Solvents used were: D1, 1.0 M sodium phosphate, pH 6.0; D3, 3.5 M lithium formate, 8.5 M urea, pH 4.0; D4, 0.8 M LiCl, 0.5 M Tris, 8.5 M urea, pH 9.0. After chromatography TLC plates were scanned using a Packard Instant Imager (Dowers Grove, IL, USA). DNA adduct levels were calculated from the adduct cpm, the specific activity of [γ-^32^P]ATP and the amount of DNA (pmol of DNA-P) used and results were expressed as DNA adducts/10^8^ nucleotides.

### 2.4 Preparation of microsomes

Hepatic microsomes from ellipticine-treated mice were isolated as described previously (Reed et al., 2018). Microsomes were isolated from 4 pooled livers of each mouse model. Protein concentration in the microsomal fraction was measured using the bicinchoninic acid protein assay with bovine serum albumin as standard. Pooled microsomal fractions were used for further experiments.

### 2.5 Enzyme activity assays and immunoblotting

The hepatic microsomal fractions were characterised for Cyp1a1 enzyme activity using Sudan I oxidation (Stiborova et al., 2002), for Cyp1a2 enzyme activity using 7-methoxyresorufin *O*-demethylation (MROD) (Burke et al., 1994), for Cyp1a1/2 enzyme activity using 7-ethoxyresorufin *O*-deethylation (EROD) (Stiborova et al., 2005) and for Cyp3a enzyme activity using testosterone 6β-hydroxylation (Stiborova, et al., 2017). POR enzyme activity was determined as described previously (Arlt et al., 2003). Western blotting analysis using 4–12% Bis-Tris gradient gels and sodium dodecyl sulphate-polyacrylamide gel electrophoresis (SDS-PAGE) were carried out as described previously (Reed et al., 2018). After migration the proteins were transferred onto polyvinylidene difluoride (PVDF) membranes and the following primary antibodies were used: anti-Cyp1a1 1:1000 (sc-20,772 (H-70), Santa Cruz Biotech); anti-Cyp3a 1:20000 (ab3572, Abcam); anti-Por 1:1000 (ab39995, Abcam), anti-Cyb5 1:750 (ab69801, Abcam); and anti-Cyb5R 1:1000 (ABIN453978, antibodies-online.com). The antibody to detect glyceraldehyde phosphate dehydrogenase (Gapdh) 1:25000 (MAB374, Chemicon) was used as loading control. The secondary horseradish peroxidase-linked antibodies were as follows: anti-goat 1:10000 (sc-2020, Santa Cruz) anti-rabbit 1:10000 (#170–5046, BioRad). The antigen-antibody complex was visualised using SuperSignal® West Pico Chemiluminescent Substrate Kit (Thermo Scientific).

### 2.6 Microsomal incubations for ellipticine-DNA adduct formation

Incubation mixtures consisted of 100 mM potassium phosphate buffer (pH 7.4). Reduced nicotinamide adenine dinucleotide (NADPH) or reduced form of nicotinamide adenine dinucleotide (NADH) (10 mM in each case), pooled hepatic microsomal fraction (0.5 mg/ml protein) from ellipticine-pretreated HRN, HBRN and WT mice, 0.1 mM ellipticine dissolved in 7.5 μl methanol and calf thymus DNA (0.5 mg) in a final volume of 750 μl. Incubations were carried out at 37 °C for 90 min (Aimova et al., 2008). Control incubations were carried out (*i*) without microsomes; (*ii*) without NADPH or NADH; (*iii*) without DNA and (*iv*) without ellipticine. After incubation, DNA was isolated by a standard phenol-chloroform extraction method. Ellipticine-DNA adduct formation was determined by ^32^P-postlabelling as described above.

### 2.7 Microsomal incubations for studying ellipticine metabolism

Incubation mixtures contained 100 mM potassium phosphate buffer (pH 7.4), NADPH or NADH (1 mM), 25 μM ellipticine dissolved in 5 μl DMSO and pooled hepatic microsomal fraction (0.5 mg/ml protein) in a final volume of 500 μl. Incubations were carried out at 37 °C for 20 min. Control incubations were carried out (*i*) without microsomes; (*ii*) without NADPH or NADH; (*iii*) without ellipticine. After incubation, 5 μl of 1 mM phenacetin (PA) in methanol was added as an internal standard. Ellipticine metabolites were extracted twice with ethyl acetate (1 ml), solvent evaporated to dryness, residues dissolved in 25 μl methanol and ellipticine metabolites separated by high performance liquid chromatography (HPLC) (5 μm Ultrasphere ODS Beckman, 4.6 mm × 250 mm preceded by a C18 guard column). The eluent was 64% methanol plus 36% of 5 mM heptane sulfonic acid in 32 mM acetic acid in water with a flow rate of 0.8 ml/min and detection was at 296 nm. Three ellipticine metabolites with the retention times of 5.7, 6.3 and 6.7 min were separated (Stiborova et al., 2004; Kotrbova et al., 2006; Stiborova et al., 2006).

### 2.8 Statistical analysis

Statistical analyses were performed with Prism GraphPad Software (Version 7.04) and *P* < .05 was considered significant.

## 3 Results

### 3.1 Protein expression of XMEs

**Fig. 2:**
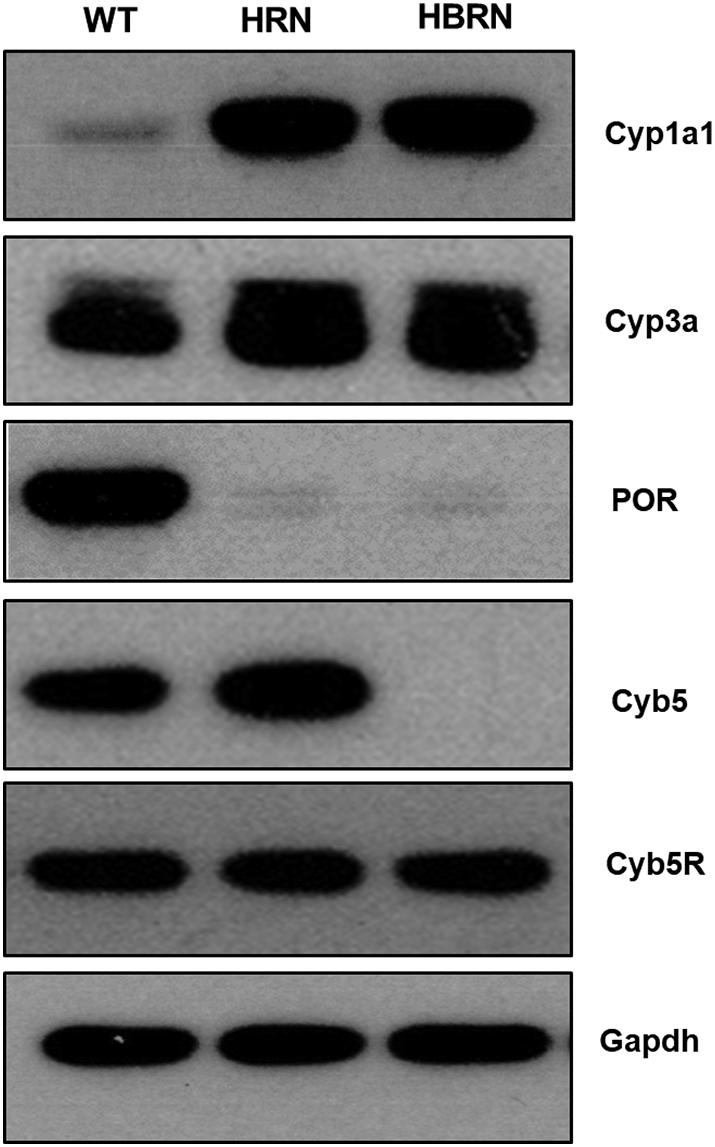
Western blot analysis of Cyp1a1, Cyp3a, POR, cytochrome *b*_*5*_ (Cyb5) and cytochrome *b*_*5*_ (Cyb5R) reductase in the pooled hepatic microsomal fractions of ellipticine-treated WT, HRN and HBRN mice (*n* = 4/group). Gapdh protein expression was used as a loading control. Representative images of the Western blotting are shown, and at least duplicate analysis was performed in separate experiments.

Expression of electron donor proteins (*i.e.* POR, Cyb5, Cyb5R) associated with the mixed-function oxidase system (*i.e.* P450) were probed for in the hepatic microsomal fractions from WT, HRN and HBRN mice exposed to ellipticine (Fig. 2). POR was expressed in the WT mice only and Cyb5 was expressed only in WT and HRN mice, as expected (Henderson et al., 2013; Reed et al., 2018). Cyb5R was expressed uniformly across all mouse lines. Cyp1a1 protein expression was greater in HRN and HBRN hepatic microsomal fractions after ellipticine treatment compared to WT. Cyp3a protein is constitutively expressed across all mouse lines but expression was increased in HRN and HBRN mice.

### 3.2 Enzyme activity of XMEs

**Fig. 3:**
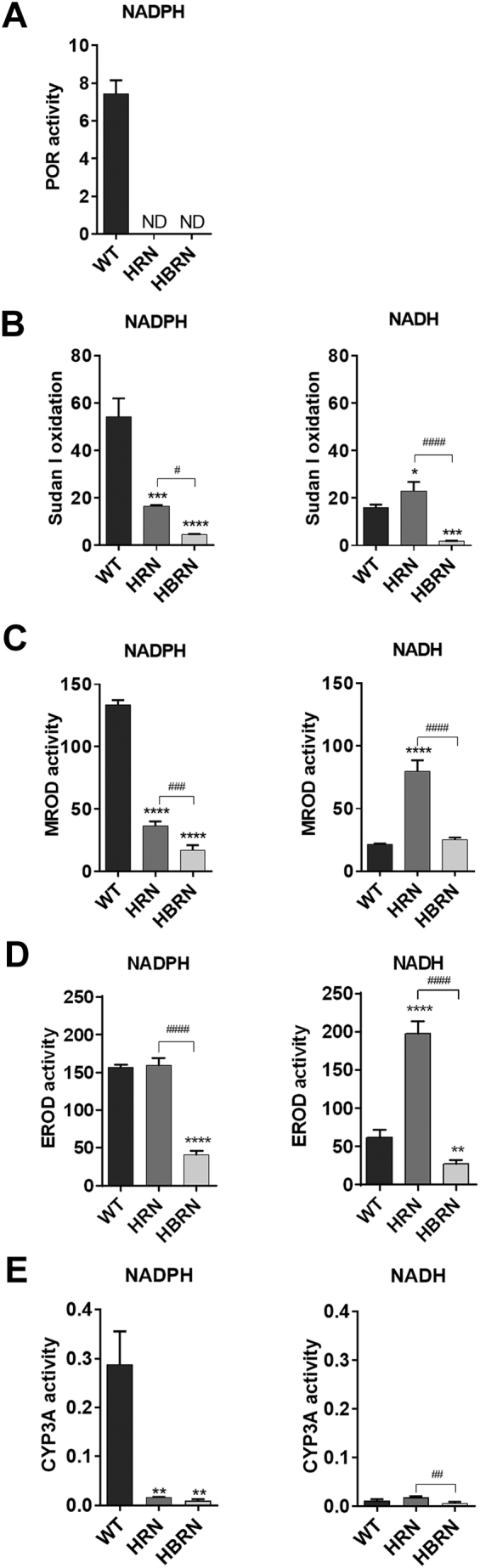
Enzyme activity in the pooled hepatic microsomal fractions of ellipticine-treated WT, HRN and HBRN mice (*n* = 4/group) using either NADPH or NADH as the enzymatic cofactor. (A) POR activity was observed as nmol of cytochrome *c*/mg/min and was only detected in microsomal fractions from WT mice. (B) Cyp1a1 activity was determined by the oxidation of Sudan I to hydroxylated metabolites with activity being observed as nmol of total C-hydroxylated metabolites/mg protein/min. (C) Cyp1a2 activity was determined using the MROD assay with activity being observed as pmol of resorufin/mg protein/min. (D) Cyp1a activity was determined using the EROD assay with activity being observed as pmol of resorufin/mg protein/min. (E) Cyp3a activity was determined by the oxidation of testosterone to hydroxylated metabolites with activity being observed as nmol of total C-hydroxylated metabolites/mg protein/min. Values are given as mean ± SD (*n* = 3) from separate experiments. Statistical analysis was performed by one-way Anova with Tukey's multiple comparison test (* = compared to WT; # = compared to HRN. * ^(#)^*P* ≤ .05 ** ^(##)^*P* ≤ .01 *** ^(###)^*P* ≤ .001 **** ^(####)^*P* ≤ .0001).

POR activity was detected in the hepatic microsomal fractions from WT mice but not in knockout animals (Fig. 3A) which is as expected given the deletion of POR in the hepatocytes of HRN and HBRN mice (Henderson et al., 2013; Arlt et al., 2015).

We used Sudan I oxidation (Fig. 3B), MROD (Fig. 3C) and EROD (Fig. 3D) as measures of Cyp1a1, Cyp1a2 and Cyp1a enzyme activity, respectively, and testosterone 6β-hydroxylation (Fig. 3E) as a measure of Cyp3a enzyme activity. When the cofactor for POR, NADPH, was used in the reaction mixture, hepatic microsomes from WT mice exhibited the highest levels of Cyp1a activity, except for in EROD where the levels of activity were equal in WT and HRN hepatic microsomal fractions (compare Fig. 3C-E). According to the Sudan I oxidation and MROD assays Cyp1a1/2 activity was significantly lower in the hepatic microsomes from HRN mice which correlated with the lack of POR activity in HRN mice relative to WT mice. Cyp1a1/2 activity in hepatic microsomes from HBRN mice was significantly lower compared to WT and HRN mice and showed the lowest level of Cyp1a1/2 activity when NADPH was present. When NADPH was used in the reaction mixture, hepatic microsomes from WT mice exhibited the highest level of Cyp3a activity, with levels being greatly lower than in the HRN and HBRN fractions.

When the cofactor for Cyb5R, NADH, was used in the reaction mixture, hepatic microsomes from HRN mice showed significantly higher Cyp1a activity than the hepatic microsomal fractions from WT and HBRN mice (Fig. 3B-D). Using MROD as a measure for Cyp1a2 activity, enzyme activity was similar in WT and HBRN mice (Fig. 3C). When EROD and Sudan I oxidation were used as a measure for Cyp1a activity hepatic microsomes from HBRN mice exhibited the lowest level of Cyp1a activity in the presence of NADH (Fig. 3B and D). When NADH was used in the reaction mixture, hepatic microsomes from HRN mice showed significantly higher Cyp3a activity than the hepatic microsomal fractions from HBRN mice but not WT mice (Fig. 3E).

### 3.3 HPLC analysis of ellipticine metabolites

**Fig. 4:**
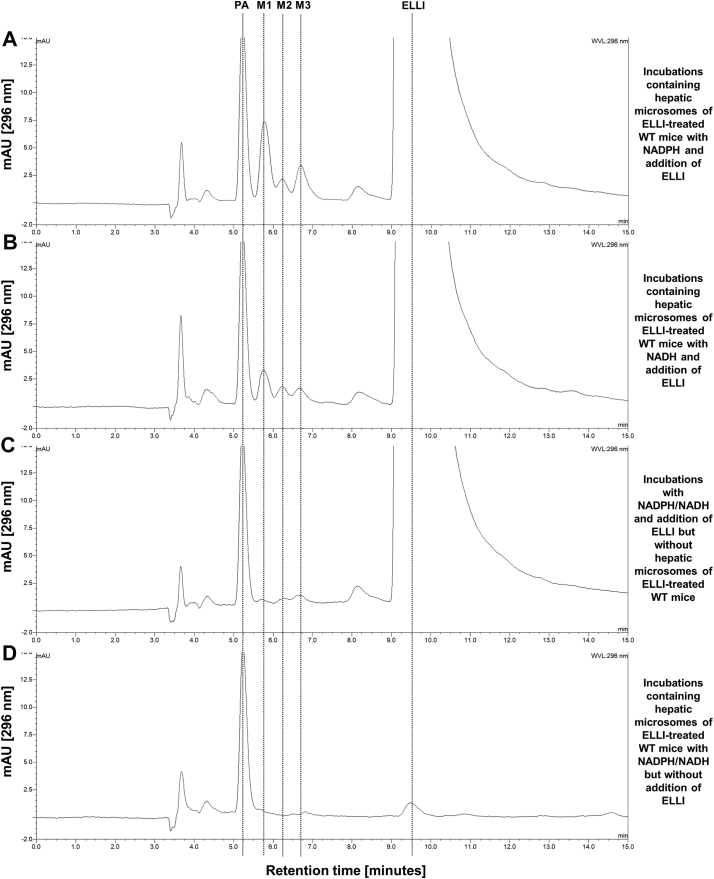
Representative HPLC chromatograms from *in vitro* incubations with pooled hepatic microsomal fractions from ellipticine-pretreated WT mice with ellipticine (ELLI) and either NADPH or NADH as cofactor. M1: 9-hydroxyellipticine; M2: 12-hydroxyellipticine; M3: 13-hydroxyellipticine. Phenacetin (PA) was used as internal standard.

**Fig. 5:**
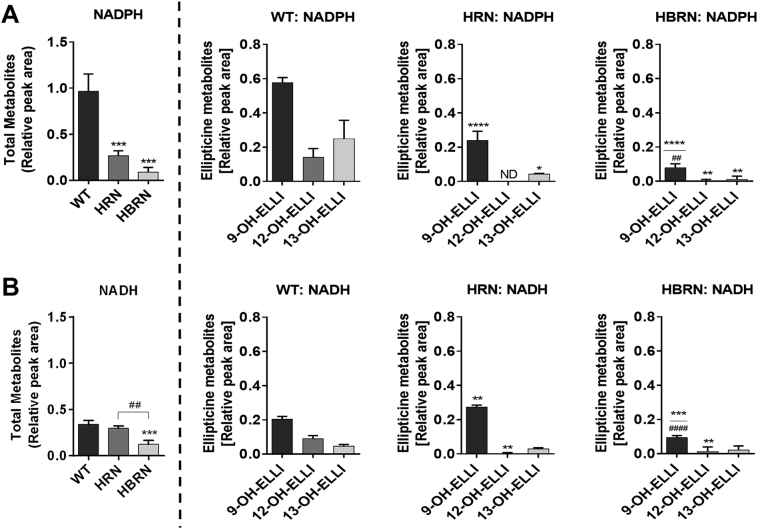
Total formation of ellipticine metabolites and formation of individual metabolites during *in vitro* incubations with pooled hepatic microsomal fractions from WT, HRN and HBRN mice (*n* = 4/group) using either NADPH (A) or NADH (B) as an enzymatic cofactor. Values are given as mean ± SD (*n* = 3) from separate experiments. Statistical analysis was performed by one-way Anova with Tukey's multiple comparison test (* = compared to WT; # = compared to HRN. * ^(#)^*P* ≤ .05 ** ^(##)^*P* ≤ .01 *** ^(###)^*P* ≤ .001 **** ^(####)^*P* ≤ .0001). ND, Not detected.

Hepatic microsomes isolated from WT, HRN and HBRN mice were incubated with ellipticine and subsequently analysed by HPLC to determine the ellipticine metabolite profile. Representative HPLC chromatograms are shown in Fig. 4. Three metabolites were formed in the microsomal incubations; 9-hydroxyellipticine (assigned peak M1), 12-hydroxyellipticine (assigned peak M2) and 13-hydroxyellipticine (assigned peak M3) (Fig. 4A and B). No metabolites were detected in control incubations without microsomes, without NADPH/NADH-generating system or without ellipticine (Fig. 4C and D). The total formation of metabolites was highest in the hepatic microsomal fraction from WT mice when NADPH was used in the reaction mix (Fig. 5A). Total ellipticine metabolite formation in hepatic microsomal fractions from WT mice was around 3-fold higher compared to when NADH was used (Fig. 5B). Hydroxylated ellipticine metabolites were identified and the structures are shown in Fig. 1.

When using NADPH as cofactor the overall formation of metabolites was significantly lower in hepatic microsomal fractions from HRN mice with production of 9-hydroxyellipticine and 13-hydroxyellipticine being significantly lower compared to WT, and 12-hydroxyellipticine not being detected (Fig. 5A). The lowest level of overall ellipticine metabolite formation was with the hepatic microsomal fractions from HBRN mice (Fig. 5A). All metabolites were detected at significantly lower amounts compared to WT hepatic microsomes and 9-hydroxyellipticine was lower compared to HRN hepatic microsomes (Fig. 5A).

When NADH was used as cofactor the overall formation of metabolites in hepatic microsomal fractions from HRN mice was not significantly different to fractions from WT with 9-hydroxyellipticine being significantly higher but 12-hydroxyellipticine being significantly lower and no significant difference in the levels of 13-hydroxyellipticine. The overall metabolite formation in hepatic fractions from HBRN mice was significantly lower than with both WT and HRN fractions (Fig. 5B) with 9-hydroxyellipticine being significantly lower compared with both WT and HRN fractions and 12-hydroxyellipticine being significantly lower compared with WT (Fig. 5B). The rate of ellipticine metabolism in the hepatic microsomal fractions correlated with the levels of Cyp1a/3a enzymatic activity and the particular enzymatic cofactor used (compare Fig. 3, Fig. 5).

### 3.4 Ellipticine-DNA adduct formation *in vitro*

**Fig. 6:**
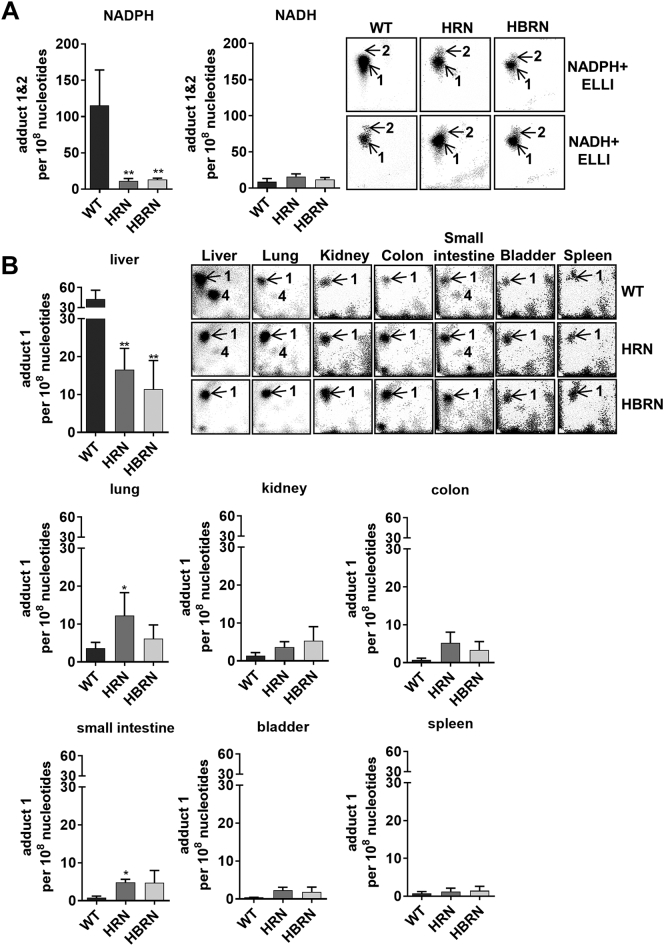
Total formation of ellipticine-DNA adducts during *in vitro* incubations with pooled hepatic microsomal fractions from WT, HRN and HBRN mice (*n* = 4/group) using either NADPH or NADH as an enzymatic cofactor (A). Values are given as mean ± SD (*n* = 3) from separate experiments. Statistical analysis was performed by one-way Anova with Tukey's multiple comparison test (* = compared to WT; ** *P* ≤ .01). Quantitative TLC ^32^P-postlabelling analysis of ellipticine-DNA adducts in organs of WT, HRN and HBRN mice treated i.p with 10 mg/kg bw ellipticine for 24 h (B). Values are given as mean ± SD (*n* = 4); DNA isolated from tissues of individual mice was analysed for each group. Statistical analysis was performed by one-way Anova with Tukey's multiple comparison test (* = compared to WT; * *P* ≤ .05). Inserts: (A) Autoradiographs of ellipticine-DNA adducts in DNA isolated from these *in vitro* incubations. (B) Autoradiographs of ellipticine-DNA adducts in liver, lung, kidney, colon, small intestine, bladder and spleen tissues in WT, HRN and HBRN mice. The origin on the TLC plate, at the bottom left-hand corners, was cut off before exposure. Adduct spots 1 and 2 are formed in deoxyguanosine residues of DNA by the ellipticine metabolites 13-hydroxy- and 12-hydroxyellipticine, respectively.

We investigated the ability of hepatic microsomes isolated from WT, HRN and HBRN mice to catalyse ellipticine-DNA adduct formation *in vitro* (Fig. 6A). The ellipticine-DNA adduct pattern obtained by ^32^P-postlabelling analysis from microsomal incubations consisted of one major and one minor adduct spot (Fig. 6A insert) (assigned adduct 1 and 2) previously detected *in vitro* and *in vivo* (Stiborova et al., 2008; Stiborova et al., 2012; Stiborova et al., 2012)*.* Because the adduct spots were incompletely separated total ellipticine-DNA adduct levels were determined. When NADPH was used the highest ellipticine-DNA adduct formation was seen in microsomal fractions from WT mice. There was significantly less DNA adduct formation with the microsomal fractions from HRN and HBRN mice (Fig. 6A). The degree of total ellipticine-DNA adduct formation in the hepatic microsomal fractions correlated with the amounts of ellipticine metabolites formed (compare Fig. 5A). When NADH was used there was no significant difference in adduct formation between any of the fractions (Fig. 6A).

### 3.5 Ellipticine-DNA adduct formation *in vivo*

The ellipticine-DNA adduct pattern obtained from *in vivo* treatments consisted of one major adduct spot (assigned adduct 1 in Fig. 6B) previously detected *in vitro* and *in vivo* (Stiborova et al., 2008; Stiborova et al., 2012; Stiborova et al., 2012). Another adduct, tentative assigned adduct spot 4, which was previously detected in ellipticine-treated rats and mice (Stiborova et al., 2003; Stiborova et al., 2007; Stiborova et al., 2008; Stiborova et al., 2014), was also generated in WT and HRN mice in selected tissues (Fig. 6B insert). As this adduct was only detectable in a few tissues (liver, lung and small intestine) in WT and HRN mice but not HBRN its level was not quantified when adduct formation was compared between tissues. Of the organs tested (liver, lung, kidney, small intestine, spleen, colon and bladder) only the liver, lung and small intestine exhibited any significant differences between the mouse models (Fig. 6B). DNA adduct formation in the livers of HRN and HBRN mice was significantly lower, by 62% and 73%, respectively, than in WT mice. In the lung and small intestine, the levels of ellipticine-DNA adducts were significantly higher in HRN mice compared to WT mice.

## 4 Discussion

Although the role of the electron donor protein Cyb5 in the metabolic activation of ellipticine has been well characterised *in vitro*, its role *in vivo* is less understood. In the present study we have used both HRN mice, which lack expression of hepatic POR, and HBRN mice, which lack expression of hepatic POR and Cyb5, to investigate the contribution of Cyb5 to the metabolic activation of ellipticine *in vivo.* These mouse models have been used recently to investigate the contribution of Cyb5 in the bioactivation of BaP *in vivo* (Reed et al., 2018). Whilst studies with hepatic microsomal fractions demonstrated the importance of Cyb5 in the metabolic activation of BaP *in vitro*, DNA adduct formation *in vivo* showed that the absence of the electron donor POR led to greatly increased levels of DNA adducts. However, the levels of BaP-DNA adducts in HBRN mice that lacked both hepatic POR and Cyb5 were significantly lower than in HRN mice demonstrating a role of Cyb5 in the metabolic activation of BaP in HRN mice. The same approach has been utilised in this study to determine the contribution of Cyb5 to the underlying mechanism of the P450-mediated metabolic activation of ellipticine.

The *in vitro* experiments in this study were carried out using pooled hepatic microsomal fractions. When NADPH was used as cofactor to examine the POR-dependent pathway, P450 enzyme activity assays clearly showed a reduction in Cyp1a activity as electron donors were lost. However, whilst significant reductions in total ellipticine metabolite and ellipticine-DNA adduct formation were found compared to WT there was no significant difference between HRN and HBRN fractions themselves, correlating with the results from Cyp3a marker activity. When the NADH-dependent Cyb5R pathway was investigated, there was higher Cyp1a enzyme activity in the microsomal fractions of HRN mice than in those of WT mice. In the microsomal fractions from HBRN mice the activity was significantly lower relative to WT mice, indicating that increased NADH-dependent activity is caused by the increased expression of Cyb5. Cyp1a activity did not correlate with total ellipticine metabolite formation as there was no significant difference between the WT and HRN fractions with only HBRN fractions showing significant reductions compared to both WT and HRN. Although this reduction in total metabolite formation would be suggestive of the contribution of Cyb5 to the activation of ellipticine, whilst all metabolites formed by HBRN fractions were reduced compared to WT, the only metabolite significantly reduced in HBRN fractions compared to HRN fractions was 9-hydroxyellipticine, a detoxication metabolite. This supports previous results in the HRN mouse with 9-hydroxyellipticine being affected by the absence of POR and P450 activity more than other metabolites (Stiborova et al., 2008). Total ellipticine-DNA adduct formation *in vitro* in the presence of NADH was lower than when NADPH was present, but not significantly different between the mouse lines.

Previous studies with reconstituted CYP1A1 and CYP1A2 showed that the presence of Cyb5 in the reaction mixture alters the resulting ellipticine metabolites profiles, with a shift from detoxication metabolites, 9-hydroxy- and/or 7-hydroxyellipticine, to metabolites that can ultimately form DNA adducts, 12-hydroxy- and/or 13-hydroxyellipticine (Kotrbova et al., 2011). These findings were echoed in studies using human CYP3A4 in Supersomes™, which found that the presence of Cyb5 in the reaction mixture also led to an increase in formation of 12-hydroxy and 13-hydroxyellipticine (Stiborova et al., 2012; Stiborova et al., 2012; Stiborova et al., 2017). In the present study we found that hepatic microsomal fractions from HBRN mice that do not possess either electron donor, metabolite formation was significantly lower than with WT fractions regardless of the cofactor used. In the hepatic microsomal fractions from HRN mice in the presence of NADH, however, the formation of 9-hydroxyellipticine was significantly higher than with microsomal fractions from WT mice whilst metabolites associated with metabolic activation were significantly lower (*i.e.* 12-hydroxyellipticine). The absence of Cyb5 in hepatic microsomal fractions from HBRN mice correlates with the findings from reconstituted P450 enzymes suggesting Cyb5 contributes to the activation of ellipticine *in vitro* (Kotrbova et al., 2011; Stiborova et al., 2012; Stiborova et al., 2012; Stiborova et al., 2017). This correlation, however, does appear to be limited to ellipticine metabolite formation. Previous studies (Poljakova et al., 2005; Kotrbova et al., 2011; Stiborova et al., 2013; Stiborova et al., 2017) found that the shift in metabolite production was coupled with an increase in *in vitro* ellipticine-DNA adduct formation. The hepatic microsomal fractions from the present study, however, found no significant difference between DNA adduct formation in HRN or HBRN hepatic microsomal fractions regardless of the enzymatic cofactor used.

The present *in vivo* results correlated with *in vitro* ellipticine-DNA adduct formation when NADPH was used as cofactor. Both HRN and HBRN mice showed significantly lower (~60–70%) hepatic ellipticine-DNA adduct formation than WT mice, but there was no significant difference between the two knockout lines. The HRN results correlate with a previous study that found a 65% reduction in hepatic DNA adduct formation in HRN mice (Stiborova et al., 2008) suggesting a greater contribution of the POR-dependent pathway to the bioactivation of ellipticine *in vivo*. *In vitro* experiments carried out in the present study with the cofactor NADH showing Cyb5R-dependent pathways contributing to ellipticine bioactivation in HRN mice correlated with *in vivo* adduct formation in the lungs and small intestine of HRN mice, suggesting that the Cyb5/Cyb5R systems contributes to ellipticine-DNA adduct formation in these extrahepatic organs of HRN mice. Ellipticine-DNA adduct formation in the extrahepatic organs of WT and HRN mice has been observed previously with HRN mice showing higher levels than WT mice, suggesting that ellipticine or its metabolites are being distributed *via* the bloodstream to organs and tissues with the metabolic capacity to oxidatively activate ellipticine (Stiborova et al., 2008). In the present study there was a trend for ellipticine-DNA adduct levels to be higher in the extrahepatic organs, *i.e.* kidney, colon and bladder, of both HRN and HBRN mice compared to WT mice, with levels of ellipticine-DNA adduct formation being significantly higher in the lungs and small intestines of HRN mice compared to WT mice. This could be due to the decreased levels of P450-mediated ellipticine metabolism in the livers of HRN and HBRN mice causing higher amounts of ellipticine to be distributed to extrahepatic organs. Levels of ellipticine-DNA adduct formation were not significantly different in any of the extrahepatic organs of HBRN mice compared to WT mice, suggesting that increased levels of activated ellipticine in the extrahepatic organs could be attributed to Cyb5 activity in the HRN mice.

The significant reduction in DNA adduct formation in the livers of HRN and HBRN mice indicates that P450 enzymes are responsible for the majority of ellipticine activation, although hepatic DNA adduct formation in HRN and HBRN mice is still detectable. It is possible that ellipticine activation still occurs due to the greater induction of hepatic P450 enzymes in HRN and HBRN mice compared to WT mice combined with expression of POR and Cyb5 in the non-parenchymal liver cells. Further investigation of this would require isolation and culture of hepatocytes and non-parenchymal liver cells from HBRN mice and exposure of these cells to ellipticine for subsequent ellipticine-DNA adduct analysis which was beyond the scope of the present study. The bioactivation of ellipticine can also be catalysed *via* P450-independent mechanisms that are not dependent on POR or Cyb5. Numerous peroxidases such as bovine LPO, human MPO, ovine COX-2 and plant HRP have previously been shown to activate ellipticine to reactive metabolites, which form two DNA adducts analogous to those generated by the P450-mediated reactions (Stiborova et al., 2007). Of the ellipticine metabolites, peroxidases predominantly form an ellipticine dimer (Stiborova et al., 2007), and generate the reactive intermediate ellipticine-13-ylium that is responsible for formation of adduct 1 (Fig. 1). Another metabolite detected was the *N*^*2*^-oxide (Fig. 1), interestingly one that is also generated by the oxidation of ellipticine by P450s and forms adduct 2 *in vitro* after the *N*^*2*^*-*oxide undergoes the Polonowski re-arrangement to form 12-hydroxyellipticine (Poljakova et al., 2005). In several tissues in ellipticine-treated WT and HRN mice (present study) we detected adduct 4 which previously has also been observed in ellipticine-treated rats (Stiborova et al., 2003; Stiborova et al., 2007; Stiborova et al., 2014) and *in vitro* studying ellipticine-DNA adduct formation recombinant human CYP3A4 expressed in Supersomes™ (Stiborova et al., 2011). However, the structure of this adduct has not yet been elucidated. Peroxidase-mediated ellipticine activation was also shown to generate two additional DNA adducts (Stiborova et al., 2007) that were, however, not detected in this study.

Studies with hepatic microsomal fractions from WT and HRN mice from mice pretreated with BaP were carried out to investigate the effect of enzyme induction on activation of ellipticine *in vitro* (Stiborova et al., 2013)*.* Microsomal incubations with ellipticine, DNA and arachidonic acid, a cofactor for COX-dependent oxidation, showed DNA adduct formation at a level similar to that when POR, Cyp1a or Cyp3a enzymes were inhibited in hepatic microsomal fractions from untreated mice. In the hepatic microsomal fractions from mice pretreated with BaP, ellipticine-DNA adduct formation with arachidonic acid was higher, very likely due to induction of COX by BaP, with no significant difference between fractions from WT and HRN mice (Stiborova et al., 2013). This participation of COX in hepatic microsomal fractions from HRN mice suggests that peroxidases could be responsible for the activation of ellipticine in the livers of HRN and HBRN mice.

In summary, this study has shown that whilst POR contributes to the bioactivation of ellipticine *in vitro,* the role of Cyb5 is still rather unclear. The *in vivo* role of Cyb5 in the activation of ellipticine in both HRN and HBRN mice appears to be less important. These findings confirm the importance of P450 enzymes in the bioactivation of ellipticine. Whilst non-parenchymal liver cells may play a role in catalysing P450-mediated bioactivation of ellipticine in HRN and HBRN mice, the presence of ellipticine-DNA adducts in the livers of HRN and HBRN mice suggests the involvement of a P450-independent bioactivation mechanism.
